# Society Meetings

**Published:** 1914-12

**Authors:** 


### SOCIETY MEETINGS

---

Brief reports and announcements of meetings of societies of Western New York, and those of general scope, are requested from Secretaries. Copy should be on hand the fifteenth of the month. Full transactions will be published at cost of composition.

---

**DOCTOR: Is your Society properly represented here? If not, please bring our standing announcement to the attention of the members.**

---

**The New York and New England Association of Railway Surgeons** held one of its most successful meetings on October 21-22, at the Hotel Astor, N. Y. City, under the presidency of Dr. C. A. Pease, Burlington, Vt.

A symposium on the Treatment of Fractures was presented and proved to be highly instructive and of great interest. The Address on Surgery, delivered by Dr. Wm. S. Bainbridge, of New York City, was deeply interesting.

Following are the officers elected for the ensuing year:—President, Dr. W. H. Marcy of Buffalo, N. Y.; First Vice-President, Dr. D. H. Lake, Kingston, Penna.; Second Vice-President, Dr. H. E. Stetson, Greenfield, Mass.; Treasurer, Dr. J. K. Stockwell, Oswego, N. Y. re-elected; Recording Secretary Dr. J. H. Reid, Troy, N. Y., re-elected; Corresponding Secretary, Dr. George Chaffee, Brooklyn, N. Y., re-elected.

The next meeting celebrating quarter century anniversary of the organization of the Association, will be held October 21-22, 1915, at the Hotel Astor, N. Y. City.

---

**The Rochester Academy of Medicine** held a special meeting November 21 at which Dr. Richard C. Cabot, of Boston, read a paper on Essentials and Non-Essentials of Physical Diagnosis.

The regular meeting of Section III. was held November 11. The program was as follows:

**a.** Changes in the Eyes Produced by Disease of the Pituitary Body, Dr. George G. Carroll.
**b.** Acromegaly and Gigantism, Dr. E. W. Mulligan.
**c.** Dispituitarism, Dr. E. G. Nugent.
**d.** Roentgenography of the Pituitary Body and the Sella Turcica, Dr. M. B. Palmer.
**e.** The Use of Pituitary Extracts in Obstetrics, Dr. J. K. Quigley.

Dr. R. R. Fitch, President; Dr. E. L. Hanes, Secretary.

---

**The Rochester Pathological Society** held its regular meeting November 5, Dr. A. T. Lytle of Buffalo reading a paper on Contract Medical Practice, an Economic Study.

---

**The Buffalo Academy of Medicine** has held the following meetings since the last notice: October 28, Stated meeting, Mendelism in its Application to the Human Species, Dr. H. H. Goddard, Director Dept. of Research, Vineland Training School, Vineland, N. J. The discussion was opened by Dr. Herman G. Matzinger.

Nov. 2, Special meeting at University of Buffalo, Medical Dept., The Electrocardiograph in Diagnosis of Heart Conditions, Dr. Thomas Lewis, London Eng.

Nov. 4, Surgical Section, Joint Tuberculosis, Dr. Leonard Ely of Leland Stanford Jr. University.

Nov. 11, Section of Obstetrics and Gynaecology, Cervical Dilatation by Hydrostatic Pressure, Dr. Wm T. Getman; Operative Treatment of Prolapse, Dr. M. D. Mann.

Nov. 18, Medical Section, the Intestinal Canal as an internal Secretory Organ; Recent Researches with Secretin, Dr. Henry R. Harrower of N. Y., Author of Practical Hormone Therapy.

---

**I. C. I. Chapter of Nu Sigma Nu** held its annual initiation and banquet November 23, the former at the Chapter House, 246 Elmwood avenue, the latter at the Lafayette.

---

**The 5th, 6th, 7th and 8th District Branches of the Dental Society of the State of New York** met in Buffalo November 19-21. Scientific papers, clinics and various social features were included.

---

**The Medical Society of the County of Monroe** adopted the following resolutions at its meeting of October 20 at Rochester:

Whereas, the Medical Society of the County of Monroe recently undertook an investigation into the prevalence and the causation of the practice of secret division of fees and granting of commissions in this county and vicinity, in an impartial and unprejudiced manner, it now seems desirable that the Society take a definite economic and moral stand on the question.

Therefore be it Resolved, that the Society hereby expresses itself definitely as being vigorously opposed to this practice in any form whatsoever, that there is no justification of this practice even on economic grounds for it seems entirely improbable that there will grow up any adequate recognition of the value of purely Diagnostic Medical Services, until this secret adjustment be abolished and that a correct and sufficient fee for such services be openly and courageously charged in its stead.

Further be it Resolved that the Society hereby inform the whole profession that our Constitution and By-Laws have always provided for the discipline of our members by censure, suspension or expulsion for such and other violations of the Principles of Medical Ethics by which all the members are bound; but that nevertheless the Society as a whole realizes that the solution of the problem lies in the ethical conduct and high moral tone of the individuals of the profession and this Medical Society of the County of Monroe therefore urges each one of the profession individually, whether a member of this Society or not, not only to refuse to engage in this practice but vigorously to oppose the same whenever and wherever possible. Charles W. Hennington, Secretary.

---

**The Rochester Medical Society** formally opened its new club house at 33 Chestnut Street, November 5. It contains an assembly room, library, reading room, dining room, a special room for women physicians, and is intended to combine the functions of a meeting place and club. The Society is a union, for these purposes, of the following organizations which will preserve their identity as originally intended: Monroe County Medical Society Rochester Pathological Society, Academy of Medicine, Homeopathic Medical Society, Hahnemann Medical Society, Hospital Medical Society, and the Blackwell Medical Society. Dr. W. B. Jones is president, Dr. F. F. Dow vice-president, Dr. D. B. Jewett secretary, and Dr. W. T. Mulligan treasurer. Drs. J. R. Culkin, E. J. Bissell, Henry Williams, W. D. Ward, W. J. Hoard, W. J. Herriman, L. L. Button and W. B. Palmer compose the Board of Directors.

The remodeling of the structure into a clubhouse was in charge of the following building committee: Dr. W. E. Ward chairman, Dr. M. B. Palmer, Dr. Wallace J. Herriman, Dr. Ralph H. Fitch, Dr. W. B. Jones and Dr. John O. Roe. The reception was in charge of of the House Committee composed of Drs. Wallace J. Herriman, T. T. Mooney and William Perrin. Dr. W. W. Percy is chairman of the Membership Committee, and Dr. John O. Roe of the Library Committee.

The property was purchased in June from A. L. Jeffreys for $35,000. Options were held on two other sites. The clubhouse project had its origin in a talk by Dr. Frank B. Tibble, secretary of the Doctors' Club of Detroit, in which he outlined the method used in organizing the Detroit Association on January 22. A feeling that the various medical associations should be housed under one roof did the rest.

---

**Value of Trees to Cities,** Stephen Smith, abstract in "Ther-Gaz.," October, 1914. Fixation of carbon dioxid and liberation of oxygen by chlorophyl under the influence of solar rays, evaporation of moisture estimated at 32,000 gallons a day for a large tree, absorption of noxious gases of putrefaction, shelter for birds which act as scavengers, are mentioned. To this may be added the value of the fire wood in an emergency, such as confronts European cities. In Paris, even the small twigs accidentally broken off or deliberately trimmed, are saved. Our western N. Y. cities are fortunate in possessing veritable forests within their limits. Except for the annoyance of petty thievery, trees in cities could be made to yield a considerable amount of fruit and nuts.

